# Response to Nitrogen Deficiency and Compensation on Physiological Characteristics, Yield Formation, and Nitrogen Utilization of Rice

**DOI:** 10.3389/fpls.2018.01075

**Published:** 2018-07-24

**Authors:** Qiangqiang Xiong, Guoping Tang, Lei Zhong, Haohua He, Xiaorong Chen

**Affiliations:** Key Laboratory of Crop Physiology, Ecology and Genetic Breeding, Ministry of Education, College of Agronomy, Jiangxi Agricultural University, Nanchang, China

**Keywords:** nitrogen deficiency and compensation effect, yield and its components, physiological characteristics, nitrogen utilization, plant growth, rice (*Oryza sativa* L.)

## Abstract

Based on the theory of ecological crop nutrient deficiency and compensation effect, the nitrogen (N) deficiency at tillering stage and N compensation at young panicle differentiation stage in rice (*Oryza sativa* L.) was selected to study. Four N treatments were treated, and the effects of N deficiency and compensation were investigated on grain yield, N uptake and utilization and the physiological characteristics of rice. The results showed that the yield per plant presented an equivalent compensatory effect. Double N compensation led to superiority in the number of effective panicle per plant, increased the activity of nitrate reductase and glutamine synthetase. The content of endogenous growth-inhibitory hormone abscisic acid (ABA) decreased in the leaves, photosynthesis was enhanced, and the number of tillers per plant increased after double N compensation. During maturation stage, the panicle dry weigh in T1 (double N compensation at young panicle differentiation stage, after N deficiency at tillering stage) was higher than that in CK1 (constant supply of N throughout different stages of growth) and the biomass per plant in T1 increased by 1.47% compared with CK1. N contents in all organs, N accumulation, and total N content were all higher in T1 during maturation stage. Moreover, N agronomic efficiency, N physiological efficiency, and N partial factor productivity were optimized for T1 and CK2 (constant N compensation at young panicle differentiation stage, after N deficiency at tillering stage) compared with CK1. This study contributes to the understanding of the physiological mechanisms underlying the compensation of N deficiency in rice.

## Introduction

To cope with growing population and limited land resources, improving rice (*Oryza sativa* L.) varieties to increase yield per unit area has become the consensus goal of global rice research. China’s Ministry of Agriculture officially approved the Chinese super rice breeding project in 1996. In 1997, Yuan proposed a technical route and vision of breeding super high-yield hybrid rice based on a “combination of morphological improvement and hybrid advantages.” Under the guidance of this technical route, China started to name super rice varieties since 2006, and a total of 156 super rice varieties (groups) have been accredited by the Chinese Ministry of Agriculture until 2016. The super rice varieties have morphological characteristics such as moderate tillering, straight flag leaves, above average short height, firm stems with lodging resistance, and large panicles with more spikelets. Moreover, these varieties exhibit superior physiological functions such as high photosynthetic efficiency, strong root activity, and coordination of “source-sink-stream.” The Chinese super rice varieties hold the record of yield. These super rice varieties are widely cultivated in the major rice-producing provinces of China, with an accumulative cultivation area of more than 70 million hectares. At present, the cultivation area has a size of more than eight million hectares per year, and especially the super hybrid rice achieves remarkable results in the double-cropping paddy fields of southern China (Wu et al., 2016). These double-cropping paddy fields of southern China are a major rice-producing region, accounting for 41.75 and 36.89% of the rice-planting area and rice yield of the whole nation, respectively (Directorate-General of Budget, Accounting, and Statistics, 2012). During recent years, super hybrid rice varieties have been popularized in the Yangtze River basin within the double-cropping region of southern China. These varieties increase both production and income, promote the renewal of rice cultivars, and significantly improve the capacity of material production and yield potential. However, these super hybrid rice varieties demand a greater amount of water and fertilizer, and they are sensitive to both water and N deficiency (Wu et al., 2008; Xue et al., 2013). Previous researchers investigated the pattern of N demand in super hybrid rice varieties (Li et al., 2012, 2014; Huang et al., 2016). A theory and technique of postponing N application was proposed, and the effects of postponed N application on photosynthesis, as well as the characteristics and yield of rice were reported (Zhang et al., 2011; Long et al., 2013). However, due to the special growing and cultivation features of super rice (Peng et al., 2009), the physiological mechanism for high yield by N manipulation in super rice remains unclear (Fu and Yang, 2012), especially in cases of improper N management during cultivation due to weather, improper management, or seasonal drought, which may cause the missing of N application during a certain developmental stage. For example, in the double-cropping region of southern China (such as the Jiangxi province), winter-to-spring droughts, summer droughts, or summer-to-fall droughts happened occasionally throughout the past few decades (Kong et al., 2010; Cai et al., 2013), causing “water breaks” in the paddy field. During these “water breaks,” it was impossible to apply fertilizer and consequently, these “water breaks” are often accompanied by “N breaks,” which requires compensation with N fertilizer when the rain resumes. Thus, it is worth to gain in-depth understanding about the formation of the N deficiency compensation effect and the underlying physiological mechanisms, as well as the changes in N uptake and utilization.

The crop growth compensation effect is a naturally existing biological phenomenon and it was first recognized when Acevedo et al. (1971) observed a rapid growing phase in plants after relief of water stress as partial compensation for loss during the stress. The physiological and ecological functions of re-watering crops has been shown to recover to a certain extent, thus partially compensating for the damage caused by drought and this was shown to have a compensatory effect for growth. The ability to compensate is different for different crops, and some crops are affected by this effect even more than they have been under proper water supply; therefore, the study of saving water and increasing output has emerged (Mu et al., 2002). Later, scientists found that compensation, as a plant physiological and ecological mechanism is an adaptive mechanism in response to deficiency of water and nutrients that has evolved during long-term environmental changes. This response helps to promote growth and production of the plant at both morphological and physiological levels (Zhou et al., 2011; Bhattacharjee and Saha, 2014). The physiological mechanisms underlying the compensation for water and nutrient deficiency mainly include dynamic changes in photosynthesis, activities of related metabolic enzymes, endogenous hormones, and tiller number (Zhao et al., 2006). Recently, pronounced compensation for N deficiency has been observed in wheat, corn, and soy (Zhai and Li, 2001, 2005; Chu and Zhang, 2010; Xing et al., 2010; Sui et al., 2013). The compensation for N deficiency has been extensively studied in wheat (Zhai and Li, 2001, 2005). This team previously reported the tillering stage of double-cropping super hybrid late rice to be sensitive to N deficiency, showing the young panicle differentiation stage as the time for effective compensation (Chen et al., 2015a,b). However, the mechanism underlying N sensitivity and compensation in double-cropping super hybrid late rice is not fully understood, and no report has been published on this topic.

Hence, this study used a double-cropping super hybrid late rice variety as testing material, and soil with low available N content was selected. N fertilizer was evenly applied at all important developmental stages, except for N deficiency treatment at tillering stage when the double-cropping super hybrid late rice was sensitive to N deficiency and N compensation with either normal compensation or double N compensation at the young panicle differentiation stage, when an effective compensation occurred. This study aimed to explore the changes in the physiological characteristics, yield, and N utilization efficiency of double-cropping super hybrid late rice in response to both N deficiency and compensation. In addition, scientific evidence was found for how to remedy inappropriate N management and guarantee stable and high yields of cultivated double-cropping super hybrid late rice, thus offering novel ecological insight into the N utilization of rice.

## Materials and Methods

### Materials

Wufengyou T025 (Wufeng A/Changhui T025, *O. sativa* L.) is a dominant double-cropping super hybrid late rice variety, which was planted in the Jiangxi province located in the double-cropping rice growing region of southern China. Wufengyou T025 is a super rice variety, certified by the crop variety approval committee of Ministry of Agriculture of China. Its yield was revealed superior during recent years in multiple places throughout Jiangxi province and its entire growth period is around 112.3 days. Agronomic traits: plant height 103.3 cm, panicle length 22.8 cm, total grains per panicle 174.6, seed setting rate 77.7%, and 1000-grain weight 22.8 g. Resistance: rice blast comprehensive index 5.5, highest panicle blast loss rate 9; bacterial blight 7; brown planthopper 9. Rice quality indicators: head rice rate 56.1%, ratio of length to width 2.9, chalky grain rate 29%, chalkiness 4.7%, gel consistency 52 mm, and amylose content 22.5 mm.

### Experimental Treatments and Cultivation Methods

Based on previous findings, double-cropping super hybrid late rice is sensitive to N deficiency at the tillering stage (Chen et al., 2015a,b). Hence, for this super hybrid late rice variety (Wufengyou T025), the tillering stage was chosen for the application of an N deficiency treatment, and N compensation was resumed at the young panicle differentiation stage. Each treatment was performed thrice with 25 pots per treatment. In 2014, the late rice was sowed on June 22, transplanted on July 23, and harvested on October 18. Fertilizer was applied at the young seedling stage, tillering stage, young panicle differentiation stage, heading stage, and milk-maturation stage on July 22, August 1, August 26, September 24, and September 30, respectively. In 2015, the late rice was sowed on June 22, transplanted on July 20, and harvested on October 22. Fertilizer was applied at the young seedling stage, tillering stage, young panicle differentiation stage, heading stage, and milk-maturation stage on July 19, July 29, August 24, September 22, and September 29, respectively. **Table 1** shows the details of N application.

**Table 1:** Experimental design of N supply.

Treatments	The total urea supply	The urea supply at different growth stage (g)				
		Young seedling stage	Tillering stage	Young panicle differentiation stage	Heading stage	Milk-maturation
CK0	0	0	0	0	0	0
CK1	4.0	0.8	0.8	0.8	0.8	0.8
CK2	3.2	0.8	0	0.8	0.8	0.8
T1	4.0	0.8	0	1.6	0.8	0.8

*CK0, N fertilizer was not supplied during all stages of growth (blank control); CK1, constant supply of N throughout different stages of growth; CK2, constant N compensation at young panicle differentiation stage, after N deficiency at tillering stage; T1, double N compensation at young panicle differentiation stage, after N deficiency at tillering stage.*

This study was conducted using soil cultivation by pots in net houses at the High-Tech Agricultural Science and Technology Park of Jiangxi Agricultural University (latitude: 28°46′N, longitude 115°50′E, altitude 48.80 m), Nanchang, Jiangxi Province of China, in 2014 and 2015. The results were similar during both years, and this study reported yields of 2014 and 2015, and analyzed the related physiological indicators and N utilization of 2015. The pots were 24.0 cm in height with an inner bottom diameter of 23.5 cm and an upper diameter of 29.0 cm. The soil was the 0∼20 cm topsoil from a field abandoned during the past few years with low levels of total N as well as available N. The physical and chemical properties of the soil of 2014 and 2015 were in **Table 2**. The soil was naturally air dried, pulverized via soil disintegrator (FT-1000A, Changzhou WIK Instrument Manufacturing Co., Ltd., China), and sieved through a 100-mesh filter. Each pot contained about 10 kg of dry soil, which was pre-drowned with water 2 weeks prior to transplantation. At the four-leaf stage, the rice seedlings that grew well and consistent were transplanted into pots at three seedlings per pot and one seedling per hole. Considering the nutrient demand of rice, 6.0 g calcium-magnesium-phosphate were added into the fertilizer, and 1.5 g KCl was added to each pot at both tillering and heading stages. Urea was applied as the N source (4.0 g per pot is equivalent to 160 kg hm^-2^, and 3.2 g per pot is equivalent to 128 kg hm^-2^. According to the experimental design, N fertilizer was mixed with the soil at the seedling stage and applied with water at all other developmental stages. The paddy was under unified management before transplantation. After transplantation, management of water and insects was done in accordance with a high-yield cultivation mode. The weather was observed after each fertilization application. The pots were moved under a shelter before heavy rain to prevent loss of fertilizer nutrients with the overflowing water, and they were moved back to the net house immediately after the rain.

**Table 2:** Physical and chemical properties of the experimental soil.

Years	Soil pH	Organic matter (g kg^-1^)	Total nitrogen (g kg^-1^)	Available nutrient (mg kg^-1^)		
				N (nitrogen)	P (phosphorus)	K (potassium)
2014	6.0	17.44	0.52	62.35	13.45	64.32
2015	5.7	17.38	0.49	64.50	11.66	75.55

### Tillering Dynamics Measurement

From the fifth day after resumption of compensatory N fertilization (N deficiency at tillering stage and fertilization was applied at the young panicle differentiation stage, i.e., several days after compensation), tillers were counted every 5 days for five consecutive counts. For each treatment group in each replicate experiment, six rice plants in a state of good and consistent growth were selected, labeled, and counted for tillers. The number of tillers was monitored until a relatively stable state.

### Net Photosynthetic Rate Measurement

From the fifth day after resumption of compensatory N fertilization (N deficiency at tillering stage and fertilization was applied at the young panicle differentiation stage, i.e., several days after compensation), the Pn was determined every 5 days for four consecutive counts. For each treatment group in each replicate experiment, three plants in a state of good and consistent growth were selected, labeled, and measured for Pn in the second leaf from the top on the stem on a sunny day between 9:00 and 11:00 am, using a CI-340 handheld photosynthesis system (CID Bio-Science, United States).

### Enzymatic Activity Related to N Metabolism Measurement

From the fifth day after resumption of compensatory N fertilization (N deficiency at tillering stage and fertilization was applied at young panicle differentiation stage, i.e., several days after compensation), enzymatic activities were measured every 5 days for five consecutive counts. For each treatment group in each replicate experiment, three plants were selected, and 0.1 g leaf sample was collected from the second leaf on the stem and the leaf samples were immediately frozen in liquid N and stored at -80°C. The activities of NR (Fatma et al., 2010) and GS (Fedorova et al., 2013) were determined with commercially available kits (COMIN, Suzhou, China).

### Endogenous Hormones Contents Measurement

On the 25th day (September 19) after resumption of compensatory N fertilization (N deficiency at tillering stage and fertilization was applied at young panicle differentiation stage, i.e., several days after compensation), 0.5 g leaf sample was collected from the second leaf on the stem of three plants for each treatment group in each replicate experiment. The leaf samples were immediately frozen in liquid N and stored at -80°C. The contents of endogenous hormones such as ABA, ZR, IAA, and GA_3_ were measured via ELISA (Yang et al., 2001).

### Dry Weigh and N Utilization Measurement

From the fifth day after resumption of compensatory N fertilization (N deficiency at tillering stage and fertilization was applied at young panicle differentiation stage, i.e., several days after compensation), dry weigh was measured every 5 days for five consecutive counts. For each treatment group in each replicate experiment, three individual plants were selected. Then the each individual plant was bagging indoor, subsequently they were through the green removing process at 105°C for 30 min. Organs such as leaf, stem and sheaths, and panicle were dried in an oven at 80°C to a constant weight, which was measured for the calculation of biomass. The N content in the crop was determined using the Kjeldahl methods (Bradstreet, 1954) and then, pulverized with a plant grinder, followed by digestion in sulphuric acid at 400°C. Then, the N contents in stem and sheaths, and leaf and seeds were measured with an automatic Kjeldahl analyzer (Kjeltec-8400, FOSS, Denmark), and the values were used to calculate the HI_N_, RE_N_, AE_N_, PE_N_, and PFP_N_.

HI_N_ = (N in grains)/(total N absorption in plant) × 100%;
RE_N_ = (total above ground plant N accumulation in the plot that received N fertilizer–total above ground plant N accumulation in the zero-N control)/(amount of N fertilizer applied) × 100%;
AE_N_ = (Grain yield in the plot that received N fertilizer–grain yield in the zero-N control)/(amount of N fertilizer applied);
PE_N_ = (Grain yield in the plot that received N fertilizer–grain yield in the zero-N control)/(total above ground plant N accumulation in the plot received N fertilizer–total above ground plant N accumulation in the zero-N control);

PFP_N_ = (Grain yield in the plot that received N fertilizer)/(amount of N fertilizer applied).

### Yield and Yield Components Measurement

After maturation, five undamaged plants per treatment group in each replicate experiment were selected and harvested to evaluate yield. The yield and effective panicle number per plant, total grain number per panicle, number of full grains, panicle length, and 1000-grain weight were measured, and the HI and seed setting rate were calculated accordingly.

Calculation method of 1000-grain weight: one thousand full-filled seeds were selected randomly for each replicate and weighed separately to get the average value;

HI=(Grainyieldperplant)/(Totalbiomassperplant).

### Statistical Analysis

Data processing station (DPS) 7.5 and OriginPro 8.5 were used to analyze data for significant differences and plotting.

Yield was the primary indicator to evaluate effects of compensation (Zhai et al., 2003). Since the crops were planted in pots in this study, the compensation effect was evaluated by yield per plant, following a previously described method whereby CI was the ratio of yield per plant after N deficiency and compensation to that of a control plant (Shen et al., 2012). The compensation effect was determined in combination with variance test results: CI > 1 with significant variance test (*P* < 0.05) results indicating over-compensation; CI equal to, or close to 1, while variance test shows insignificance (*P* < 0.05). This allows consideration of equivalent compensation; CI < 1 with a significant variance test result (*P* < 0.05) that indicates inadequate compensation.

## Results

### Yield and Yield Components and Tiller Number

An analysis of variance indicated (**Table 3**) that N deficiency and compensation treatments (A) was significant (*P* < 0.01), the difference between years of treatments (Y) was also significant (*P* < 0.05), but the difference in yield was not significant for different years and the trends of changes in different treatments were basically consistent in different years.

**Table 3:** Analysis of variance of yield under nitrogen deficiency and compensation.

SV	DF	SS	MS	*F*-value	*F*_0.05_	*F*_0.01_
Replication	4	16.33	4.08	1.02^ns^	2.71	4.07
Treatments	7	1346.84	192.41	47.88^∗∗^	2.36	3.36
Year (Y)	1	20.87	20.87	5.19^∗^	4.2	7.64
A	3	1322.14	440.71	109.67^∗∗^	2.95	4.57
Y × A	3	3.83	1.28	0.32^ns^	2.95	4.57
Error	28	112.52	4.02			
Total	39	1475.69				

*CK0, N fertilizer was not supplied during all stages of growth (blank control); CK1, constant supply of N throughout different stages of growth; CK2, constant N compensation at young panicle differentiation stage, after N deficiency at tillering stage; T1, double N compensation at young panicle differentiation stage, after N deficiency at tillering stage. SV, source of variation; DF, degrees of freedom; SS, sum of square; MS, mean square; F, variance ratio; Y, year; A, nitrogen deficiency and compensation treatments; ^∗^P < 0.05; ^∗∗^P < 0.01; ns, not significantly different.*

The yield and yield components varied among different treatment groups in 2014 (**Table 4**). The yield per plant, effective panicle per plant, and 1000-grain weight were relatively low in CK0, while T1 had the highest yield per plant, effective panicles per plant, and HI. The yield per plant in CK0 was significantly (*P* < 0.05) different from that in CK1, CK2, and T1. The yield per plant in T1 increased by 11.73% compared with that of CK1, but the difference was not statistically significant (*P* < 0.05), thus T1 showed an equivalent compensation effect (CI = 1.12). HI was 7.42% significantly (*P* < 0.05) higher in T1 compared with CK1. The number of effective panicle of T1 was significantly (*P* < 0.05) increased by 34.78 and 19.23%, respectively, compared with CK1 and CK2. Compared with CK1, the 1000-grain weight in CK2 and T1 had increased by 2.81 and 1.05%, respectively. These results indicated that for this double-cropping super hybrid late rice, double N compensation after N deficiency would rendere visible compensation in terms of yield per plant, effective panicle per plant and HI. However, the effects on panicle length, total grain number per panicle, and seed setting rate were not significant (*P* > 0.05). Double N compensation after N deficiency improved the yield per plant in the double-cropping super hybrid late rice, particularly by increasing the effective panicles after tillering.

**Table 4:** Effect of nitrogen deficiency and compensation on yield and its components in double-cropping super hybrid late rice.

Years	Treatments	Effective panicle per plant	Panicle length (cm)	Number of total grains	Seed setting rate^a^ (%)	1000-grain weight (g)	Yield per plant (g)	HI^b^ (%)	CI^c^
2014	CK0	2.80c	21.54b	170.36b	91.73b	21.93b	9.19c	60.71b	0.43
	CK1	4.60b	24.31a	218.50a	95.91a	22.80ab	21.39ab	62.16b	1
	CK2	5.20b	22.22b	173.15b	93.99ab	23.44a	19.41b	62.10b	0.91
	T1	6.20a	22.94ab	194.79b	92.49b	23.04a	23.90a	66.77a	1.12
2015	CK0	3.00c	22.47a	176.60a	91.36b	21.90b	9.91c	56.62b	0.42
	CK1	5.60b	23.63a	201.75a	94.58a	22.84a	23.63ab	59.07ab	1
	CK2	5.20b	23.92a	187.79a	95.31a	22.99a	21.25b	60.11a	0.9
	T1	6.40a	22.96a	190.80a	93.34a	22.78a	24.87a	61.55a	1.05

*CK0, N fertilizer was not supplied during all stages of growth (blank control); CK1, constant supply of N throughout different stages of growth; CK2, constant N compensation at young panicle differentiation stage, after N deficiency at tillering stage; T1, double N compensation at young panicle differentiation stage, after N deficiency at tillering stage. Within each year, values in the same column show mean via the Tukey’s multiple range test, and different letters indicate that the means were statistically different (P < 0.05), n = 4; Data are averages observed for two study years (n = 6, 2 years × 3 replicates). ^a^Seed setting rate = number of filled grains per panicle/number of total grains per panicle. ^b^HI = total grain weight per plant (dry weigh)/total above ground biomass per plant (dry weigh). ^c^CI = yield per plant/yield per plant of CK1.*

The yield per plant in CK2 and T1 of 2015 after N compensation were significantly higher (*P* < 0.05) than those in CK0. T1 had the highest yield per plant, which was 5.25% higher than that in CK1. It shows an equivalent compensation effect (CI = 1.05). For the yield components, the effective panicle per plant in T1 were significantly (*P* < 0.05) higher compared with the other treatment groups. T1 was 14.29% higher than those in CK1. The number of total grains per panicle was the highest in CK1 and the lowest in CK0. The 1000-grain weight in CK1 was not significantly (*P* > 0.05) different from that in CK2 and T1. The HI was maximal in T1 and it was 4.20% higher than that in CK1 with no significant difference (*P* > 0.05) (**Table 4**).

In summary, the yield and yield components in each treatment group were comparable between both evaluated years. In the double-cropping super hybrid late rice, double N compensation after N deficiency compensated the yield. In terms of yield components, this compensation relied on an increase in the number of effective panicle per plant. Yield per plant and CI were similar in CK1 and T1 treatments in 2014 and 2015 years. Thus, the advantage of double N compensation after N deficiency will remedy inappropriate N management and guarantee stable and high yields of cultivated in double-cropping super hybrid late rice.

**FIGURE 1:**
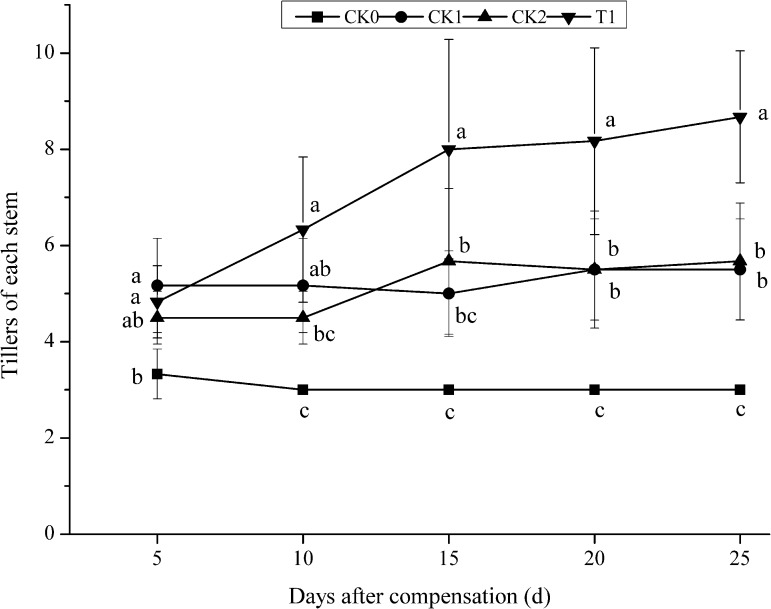
Dynamics of tillering under nitrogen deficiency and during compensation in double-cropping super hybrid late rice during the year 2015; The column data in figure is average and the short lines represents the mean square deviation, values in the same column show means via the Tukey’s multiple range test, and different letters indicate that the means were statistically different (*P* < 0.05), *n* = 6.

In the early stage of N compensation at the young panicle differentiation stage after N deficiency at tillering stage, the number of tillers per plant was higher in CK1 than in the other treatment groups (CK0, CK2, and T1) (**Figure 1**). Along with plant development and growth, the number of tillers per plant maintained unchanged in CK0. Whereas, the tiller number per plant increased in CK2 and T1 after N compensation. After 10 days compensation, the tiller number per plant in T1 was 40.00 and 21.15% higher compared with CK2 and CK1, respectively. There was no significant difference (*P* > 0.05) between T1 and CK1, but T1 and CK2 showed significant difference (*P* < 0.05). Furthermore, it remained significantly (*P* < 0.05) higher level compared with CK1 and CK2 until the late stage of compensation when the tiller numbers did not change any more. These results indicated that double N compensation promoted tillering in double-cropping super hybrid late rice and demonstrated the effect of N deficiency and compensation on rice tillering.

### Net Photosynthetic Rate

The Pn varied between N deficiency at tillering stage and later compensation in double-cropping super hybrid late rice (**Table 5**). Pn of CK0 was the lowest. After 5 days compensation, the Pn of CK2 and T1 were significantly (*P* < 0.05) higher than that in CK0. The Pn of CK1 was not significantly different (*P* > 0.05) from that in CK2 and T1. As the plants grew, the Pn of CK0 declined. After 10 days compensation, the Pn of CK1 and T1 increased. Then started declining after 15 days compensation, the Pn of T1 and CK1 decreased by 2.54 and 9.33%, respectively. The rate of decrease was lower in T1 compared with CK1. After 20 days compensation, the Pn of CK2 and T1 were significantly (*P* < 0.05) higher than those in CK0 and CK1. At the heading stage, the Pn of CK2 and T1 were still higher than those in CK1, but the differences were not significant (*P* > 0.05). These results indicated that the double-cropping super hybrid late rice retained a relatively high Pn after double N compensation, which demonstrated a prominent compensation effect for N deficiency.

**Table 5:** Dynamics of net photosynthetic rate in the second leaf from the top under the treatment of nitrogen deficiency and compensation in 2015.

Treatments	5 days after compensation (mmol m^-2^ s^-1^)	10 days after compensation (mmol m^-2^ s^-1^)	15 days after compensation (mmol m^-2^ s^-1^)	20 days after compensation (mmol m^-2^ s^-1^)	Heading stage (mmol m^-2^ s^-1^)
CK0	23.20b	22.20c	20.57b	19.23c	21.93b
CK1	30.77a	32.47a	29.70a	26.53b	28.13a
CK2	30.50a	29.70a	29.73a	29.57a	29.23a
T1	30.26a	32.30a	31.50a	29.80a	29.10a

*CK0, N fertilizer was not supplied during all stages of growth (blank control); CK1, constant supply of N throughout different stages of growth; CK2, constant N compensation at young panicle differentiation stage, after N deficiency at tillering stage; T1, double N compensation at young panicle differentiation stage, after N deficiency at tillering stage. In 2015, values in the same column show means of the Tukey’s multiple range test, and different letters indicate that the means were statistically different (P < 0.05), n = 4. Data are averages observed for the study year of 2015 (n = 3, 1 year × 3 replicates).*

### Enzymatic Activity Related to N Metabolism

**FIGURE 2:**
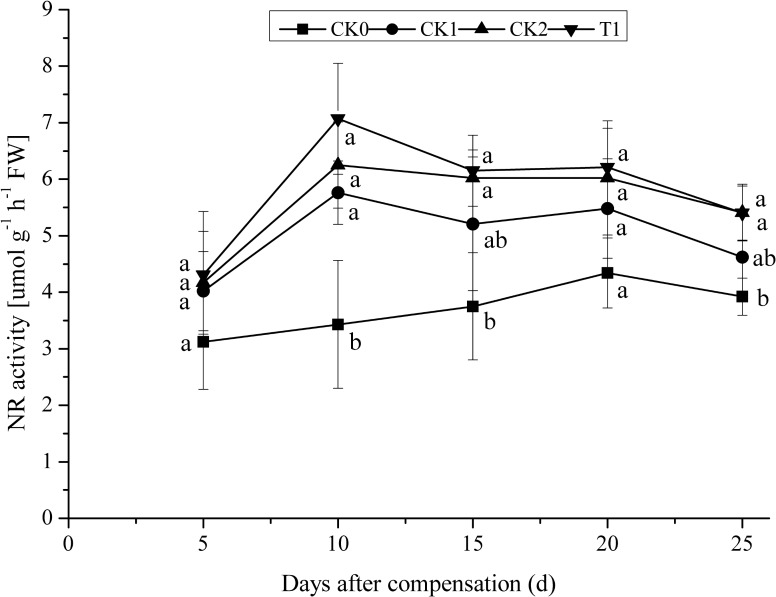
Dynamics of NR activity under the treatment of nitrogen deficiency and compensation in double-cropping super hybrid late rice during the year 2015; The column data in figure is average and the short lines represents the mean square deviation, values in the same column show means via the Tukey’s multiple range test, and different letters indicate that the means were statistically different (*P* < 0.05), *n* = 3.

After N deficiency and N compensation, the activity of NR first increased to the maximum and then declined (**Figure 2**). After 10 days compensation, NR activities in CK1, CK2, and T1 reached the maximum. NR activities in CK2 and T1 were 8.51 and 22.74% higher than in CK1. As the plants grew, NR activities in CK1, CK2, and T1 slightly decreased. NR activities in CK2 and T1 were maintained at a relatively higher level compared with those in CK1. After 25 days compensation, NR activities in CK2 and T1 were 17.10 and 16.88% higher than in CK1, respectively. No significant difference (*P* > 0.05) between CK2, T1, and CK1. These results indicate that double N compensation after N deficiency, could improve NR activity to some extent in double-cropping super hybrid late rice. Consequently, increasing the capacity of N uptake and utilization in rice provides a strong improvement of N use efficiency of rice.

**FIGURE 3:**
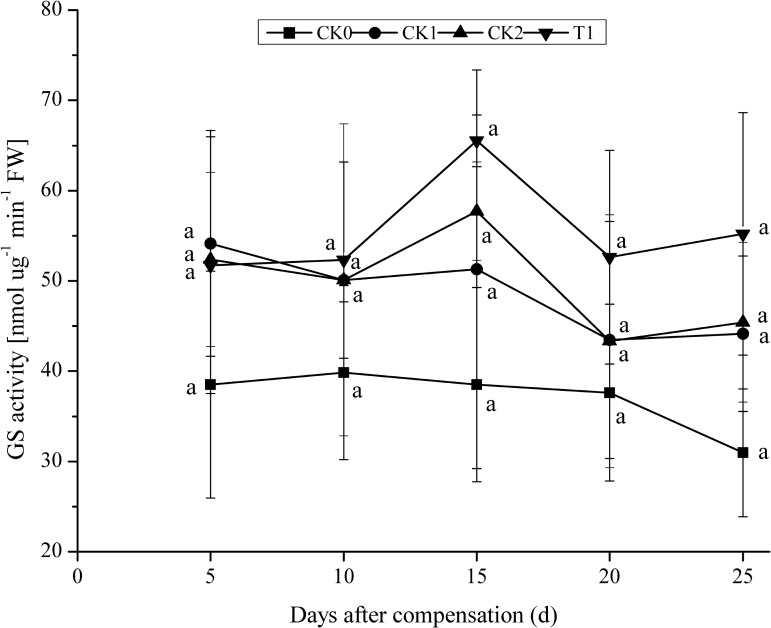
Dynamics of GS activity under the treatment of nitrogen deficiency and compensation in double-cropping super hybrid late rice during the year 2015; The column data in figure is average and the short lines represents the mean square deviation, values in the same column show means via the Tukey’s multiple range test, and different letters indicate that the means were statistically different (*P* < 0.05), *n* = 3.

After 5 days N compensation, CK1 showed maximal GS activity. As the plants grew, after 10 days compensation, GS activity in CK2 and T1 presented a pattern of initial increase, followed by a decline (**Figure 3**). After 15 days compensation, GS activities in CK2 and T1 reached a maximum, and they were 12.51 and 27.74% higher compared with CK1. No significant difference (*P* > 0.05) between CK2, T1, and CK1. After 25 days compensation, T1 still showed high GS activity, which was 21.58 and 25.08% higher than that in CK2 and CK1. No significant difference (*P* > 0.05) between CK2, CK1, and T1. Generally, GS activity in CK1, CK2, and T1 presented a pattern of first rising and then declining, whereas GS activity in CK0 declined all the way. These results indicate that GS activity increased rapidly in a short time under the treatment of N compensation after N deficiency. GS activity is higher than that of other treatments to some extent after double N compensation. These results demonstrated a certain compensation effect for N deficiency.

### Endogenous Hormones Contents

After 25 days N compensation (September 19) in double-cropping super hybrid late rice, ABA contents in CK2 and T1 with N deficiency and compensation were significantly lower (*P* < 0.05) compared with CK0 and CK1 (**Table 6**). The contents of GA_3_ was maximal in T1, and the levels were significantly higher (*P* < 0.05) than in CK0. The contents of IAA was maximal in T1, and the levels were significantly higher (*P* < 0.05) than in CK0 and CK1. The contents of ZR were higher in CK0 and CK1. The content of ZR was lower in CK2 and T1. There was a significant difference (*P* < 0.05) between CK0, CK1 and CK2, T1. The ratio of total growth-promoting hormones (GA_3_ + IAA + ZR) to growth-inhibitory hormone (ABA) in CK2 and T1 was significantly (*P* < 0.05) higher than that in CK0 and CK1. These results indicate that the endogenous hormones were optimized in double-cropping super hybrid late rice after N deficiency and N compensation. The levels of pro-growth hormones were markedly increased, while the anti-growth hormone was significantly reduced, and the changes were prominent in T1 that received double-amount N compensation.

**Table 6:** Differences in endogenous hormones in leaves 25 days after nitrogen deficiency and compensation (September 19) in double-cropping super hybrid late rice in 2015.

Treatments	ABA (ng g^-1^ FW)	GA_3_ (ng g^-1^ FW)	IAA (ng g^-1^ FW)	ZR (ng g^-1^ FW)	(GA_3_ + IAA + ZR)/ABA
CK0	91.71a	9.45c	35.09c	10.22a	0.60d
CK1	94.84a	11.30a	46.89b	10.07a	0.72c
CK2	74.43c	11.91a	45.29b	9.23b	0.89b
T1	79.87b	12.12a	67.57a	9.04b	1.11a

*CK0, N fertilizer was not supplied during all stages of growth (blank control); CK1, constant supply of N throughout different stages of growth; CK2, constant N compensation at young panicle differentiation stage, after N deficiency at tillering stage; T1, double N compensation at young panicle differentiation stage, after N deficiency at tillering stage. In 2015, values in the same column show mean via the Tukey’s multiple range test, and different letters indicate that the means were statistically different (P < 0.05), n = 4. Data are averages observed for the study year of 2015 (n = 3, 1 year × 3 replicates)*

### Dry Weigh Accumulation and Distribution

Dry weigh in the stem and sheaths as well as the leaves was minimal in CK0 and maximal in CK1 (**Table 7**). The dry weigh in the stem and sheaths as well as the leaves was not significantly different among CK1, CK2, and T1. At heading stage, dry weigh in the panicle was maximal in CK1, followed by T1 and CK2 There were not significantly (*P* < 0.05) different between CK1 and CK2 or T1. At maturation stage, dry weigh in the panicle was maximal in T1, followed by CK1. Panicle dry weigh in T1 was 5.82% higher than in CK1, but the difference was not significant (*P* > 0.05). These results indicated that double N compensation after N deficiency compensated the production of dry matter in double-cropping super hybrid late rice. The dry weigh in the stem and sheaths at maturation stage was decreased compared with that at the heading stage, which was related to the export of stem-sheaths matter. The rates of stem-sheaths matter export in CK0, CK1, CK2, and T1 were 27.32, 23.82, 30.97, and 27.15%, respectively. The export rates in CK2 and T1 were significantly higher (*P* < 0.05) than those in CK1. It indicates optimization of assimilate transport and distribution to the panicle after N compensation after N deficiency.

**Table 7:** Effects of nitrogen deficiency and compensation on dry matter accumulation and distribution in different organs in 2015.

Treatments	Young panicle differentiation stage (g)		Heading stage (g)			Maturation stage (g)			Stem and sheath output rate^a^ (%)
	Stem and sheath	Leaf	Stem and sheath	Leaf	Panicle	Stem and sheath	Leaf	Panicle	
CK0	2.71a	1.63b	7.32b	2.00b	1.91b	5.32c	2.03c	10.14c	27.32b
CK1	4.28a	3.03a	14.15a	4.17a	3.75a	10.78a	4.66ab	23.37ab	23.82c
CK2	3.90a	2.70a	12.85a	3.48a	3.31a	8.87b	3.82b	20.51b	30.97a
T1	4.26a	2.79a	13.63a	4.37a	3.25a	9.93ab	4.72a	24.73a	27.15b

*CK0, N fertilizer was not supplied during all stages of growth (blank control); CK1, constant supply of N throughout different stages of growth; CK2, constant N compensation at young panicle differentiation stage, after N deficiency at tillering stage; T1, double N compensation at young panicle differentiation stage, after N deficiency at tillering stage. In 2015, values in the same column show the means via the Tukey’s multiple range test, and different letters indicate that the means were statistically different (P < 0.05), n = 4. Data are averages observed for the study year of 2015 (n = 3, 1 year × 3 replicates). ^*a*^Stem and sheath output rate = [stem and sheaths at the heading stage (dry weigh) – stem and sheaths at the maturation stage (dry weigh)]/stem and sheaths at the heading stage (dry weigh).*

**FIGURE 4:**
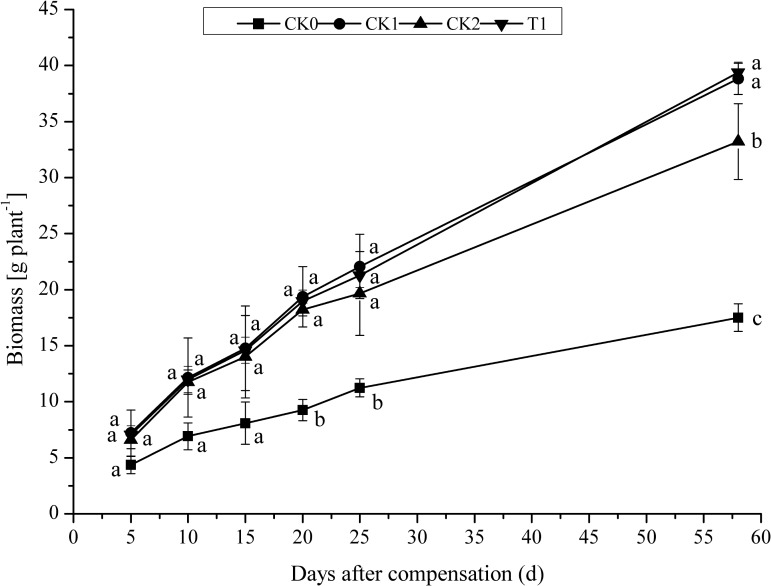
Effect of nitrogen deficiency and compensation on biomass per plant in double-cropping super hybrid late rice during the year 2015; The column data in figure is average and the short lines represents the mean square deviation, values in the same column show means via the Tukey’s multiple range test, and different letters indicate that the means were statistically different (*P* < 0.05), *n* = 3.

After 5 days N compensation, biomass per plant in CK0 was minimal throughout all developmental stages (**Figure 4**). As the plants grew, biomass per plant increased in all treatment groups. After 10 days compensation, biomass per plant rapidly increased in CK1, CK2, and T1. After 25 days compensation (heading stage), biomass per plant in T1 was 3.67% lower compared with CK1. No significant difference (*P* > 0.05) between CK1 and T1. However, biomass per plant in T1 was 1.47% higher than in CK1 at maturation stage. Biomass per plant in CK1 and T1 was significantly (*P* < 0.05) higher than that of CK0 and CK2. These results indicated that N fertilizer promoted biomass per plant accumulation in the double-cropping super hybrid late rice. With the application of the same amount of N fertilizer, biomass per plant increased rapidly in early compensation of N. Nevertheless, the difference gradually decreased as plants grew between CK1 and T1. After 58 days compensation (maturation stage), biomass per plant was maximal in T1, suggesting that the double amount of N compensation after N deficiency produced a compensatory effect on biomass accumulation in double-cropping super hybrid late rice, thus guaranteeing yield increase.

### N Contents, Distribution, and Utilization

CK0 had the lowest N contents in all organs (spike, stem and sheaths, and leaf) at maturation stage among all treatment groups (**Table 8**). CK2 and T1 had significantly (*P* < 0.05) higher N contents in the spike, stem and sheaths, and leaves compared with CK0. There was no significant difference in N contents of all organs between CK2 and CK1. The N contents in the stem and sheaths of T1 was significantly (*P* < 0.05) higher than that of CK1. N contents in the spike and leaves of T1 were not significantly different from those of CK1. As the amount of total N supply increased, N accumulation increased in all organs (spike, stem and sheaths, and leaf), and total N accumulation increased markedly in CK1 and T1. Moreover, T1 had the highest spike N accumulation and total N accumulation. With the application of equal amounts of N fertilizer, total N content in T1 was 5.24% higher than that in CK1. These results indicate that the resumption of N compensation after N deficiency improved N uptake and distribution in double-cropping super hybrid late rice. This compensatory effect was significant (*P* < 0.05).

**Table 8:** Effects of nitrogen deficiency and compensation on N contents and accumulation in different organs in 2015.

Treatments	N contents (%)			N accumulation (g)			Total N (g)
	Spike	Stem and sheaths	Leaf	Spike	Stem and sheaths	Leaf	
CK0	9.10b	3.10c	7.20b	0.090c	0.015c	0.014b	0.121c
CK1	14.50a	4.80b	9.70a	0.342a	0.051ab	0.045a	0.439a
CK2	14.00a	5.20b	9.40a	0.297b	0.049b	0.037a	0.383b
T1	14.30a	6.10a	9.80a	0.357a	0.059a	0.046a	0.462a

*CK0, N fertilizer was not supplied during all stages of growth (blank control); CK1, constant supply of N throughout different stages of growth; CK2, constant N compensation at young panicle differentiation stage, after N deficiency at tillering stage; T1, double N compensation at young panicle differentiation stage, after N deficiency at tillering stage. In 2015, values in the same column show the means via the Tukey’s multiple range test, and different letters indicate that the means are statistically different (P < 0.05), n = 4. Data are averages observed for the study year of 2015 (n = 3, 1 year × 3 replicates).*

After N deficiency and N compensation, RE_N_ was maximal in T1, followed by CK1 (**Table 9**). RE_N_ in T1 increased by 7.27% compared with CK1. With equal amounts of N application, AE_N_ in T1 was 9.02% higher than in CK1. HI_N_ in CK1 was not significantly different from those in CK2 and T1. PE_N_ in CK2 and T1 were 2.08 and 1.02% higher than in CK1, respectively, but it was not statistically significant (*P* > 0.05). With the application of equal amounts of N fertilizer, PFP_N_ was not significantly different between CK1 and T1, whereas PFP_N_ in CK2 was 15.83% higher than in CK1.

**Table 9:** Effects of nitrogen deficiency and compensation on N absorption and utilization efficiency of double-cropping super hybrid late rice in 2015.

Treatments	RE_N_^a^ (%)	AE_N_^b^ (kg kg^-1^)	PE_N_^c^ (kg kg^-1^)	PFP_N_^d^ (kg kg^-1^)	HI_N_^e^ (%)
CK0	0	0	0	0	74.80b
CK1	52.30a	22.50a	43.25a	38.74a	78.10a
CK2	52.00a	23.15a	44.15a	43.38a	77.60a
T1	56.10a	24.53a	43.69a	40.78a	77.00a

*CK0, N fertilizer was not supplied during all stages of growth (blank control); CK1, constant supply of N throughout different stages of growth; CK2, constant N compensation at young panicle differentiation stage, after N deficiency at tillering stage; T1, double N compensation at young panicle differentiation stage, after N deficiency at tillering stage. In 2015, values in the same column show means via the Tukey’s multiple range test, and different letters indicate that the means were statistically different (P < 0.05), n = 4. ^a^HI_N_, N harvest index = (N in grains)/(total N absorption in plant) × 100%. ^b^RE_N_, N recovery efficiency = (total above ground plant N accumulation in the plot that received N fertilizer–total above ground plant N accumulation in the zero-N control)/(amount of N fertilizer applied) × 100%. ^c^AE_N_, N agronomic efficiency = (Grain yield in the plot that received N fertilizer–grain yield in the zero-N control)/(amount of N fertilizer applied). ^d^PE_N_, N physiological efficiency = (Grain yield in the plot that received N fertilizer–grain yield in the zero-N control)/(total above ground plant N accumulation in the plot received N fertilizer–total above ground plant N accumulation in the zero-N control). ^e^PFP_N_, N partial factor productivity = (Grain yield in the plot that received N fertilizer)/(amount of N fertilizer applied; Data are averages observed for the study year of 2015) (n = 3, 1 year × 3 replicates).*

## Discussion

Zhai and Li (2001, 2005) pointed out that N deficiency during over-wintering would significantly reduce biomass production and grain yield in winter wheat, while resumption of N supply at the jointing stage may compensate the yield of winter wheat. Comparing the effects of N deficiency at different developmental stages on the growth of winter wheat, N deficiency have the largest impact on yield at both over-wintering period and shooting stage, whereas the impact of N deficiency at later stages is minimal. Chen et al. (2015a,b) reported that yield per plant of double-cropping super hybrid rice was significantly reduced under N deficiency at tillering stage and young panicle differentiation stage, while N deficiency had little impact on yield after both seedling stage and heading stage. Moreover, after N deficiency at tillering stage or young panicle differentiation stage, double N compensation led a higher yield compared with a constant supply of N, which demonstrated a typical compensatory effect for N deficiency. This study showed that, in 2014 and 2015, the yield per plant in double-cropping super hybrid late rice with double N compensation at young panicle differentiation stage after N deficiency at tillering stage (T1) was higher than plants that received N fertilizer throughout all developmental stages (no N deficiency, CK1). The results were not significantly different between T1 and CK1 for both years, and the CI indicated an equivalent compensation effect. These results are consistent with previous studies (Chen et al., 2015a,b). This study demonstrates the existence of a certain compensation for N deficiency in double-cropping super hybrid late rice, and also confirms the young panicle differentiation stage as an effective compensation period for N deficiency in double-cropping super hybrid late rice (Chen et al., 2015a,b). It should be noted that Zhong et al. (2007) from the rice research institute at the academy of agricultural sciences in Guangdong of China, have established a “Tri-control” rice fertilization system, controlling fertilization, seedlings and insects, in which the controls of total N supply and base-tiller N supply were highlighted. After determination of the amount of total N supply, N was applied at a fix rate to the total N supply at each stage. Base fertilizer accounted for 35–40% of total N supply, while the middle tillering stage (from transplantation till the middle point of early young panicle differentiation stage) accounted for about 20%, the early young panicle differentiation stage accounted for 35–40% and the heading stage accounted for 5–10%. Thus, in short supply of total N, the “Tri-control” fertilization methods emphasize the actions of reducing N supply at tillering stage and postpones N application with increased N supply from panicle differentiation stage onwards, to ensure a high and stable yield of rice. In this study, the resumption of N supply at young panicle differentiation stage after N deficiency at tillering stage induced equivalent compensation and our findings support the core-technique of N application according to the “Tri-control” system. In this study, double N compensation at young panicle differentiation stage after N deficiency at tillering stage was actually postponing N application. Postponed N application did not reduce the yield to a significant extent, which suggesting that (1) N application can be postponed during the cultivation of double-cropping super hybrid late rice, but not later than the heading stage. (2) Inadequate N supply at the young panicle differentiation stage and before due to various reasons (such as drought), can be compensated as late as the panicle differentiation stage, still guaranteeing yield. In practice, the effect of topdressing depends on the status of the seedlings and on soil fertility. Therefore, special attention should be paid to the application of panicle fertilizer during the cultivation of double-cropping super hybrid late rice.

The yield per plant of rice was determined by four factors: the number of effective panicles per plant, number of grains per panicle, seed setting rate, and 1000-grain weight (Li et al., 2009). Chen et al. (2008) proposed that increasing the ratio of N supply at the young panicle differentiation stage could optimize the outcome of panicle development. In this study, after normal N compensation or double N compensation at young panicle differentiation stage after N deficiency at tillering stage in double-cropping super hybrid late rice, the number of effective panicle per plant, HI and 1000-grain weight increased to different extents, in particular, the number of effective panicle per plant showed a remarkable increase. The number of effective panicle per plant in T1 was significantly higher than that in CK1 in both years, whereas N deficiency and compensation had little impact on the number of grains per panicle or on the seed setting rate. We further studied the cause for this increase in effective panicle per plant double N compensation at young panicle differentiation stage after N deficiency at tillering stage (T1). Based on tillering dynamics, we found that although the number of tillers in T1 was lower than that in CK1 at an early stage, double N compensation induced rapid tiller growth and these young tillers displayed significant advantages in survival. Therefore, the number of effective panicle per plant in T1 was superior to that in CK2. In summary, in double-cropping super hybrid late rice, the compensation on yield per plant via N re-application at young panicle differentiation stage for N deficiency at tillering stage mainly depends on the increase in the number of effective panicle per plant. In addition, it should be noted that growth compensation takes time (Zhai and Li, 2001, 2005; Zhao et al., 2006; Chu and Zhang, 2010; Xing et al., 2010; Sui et al., 2013), thus, compensation in double-cropping super hybrid late rice has limitations. During cultivation, we can benefit from compensation for loss due to bad weather or improper management, more importantly the management during the whole course of production needs to be optimized. Overall, panicle fertilizer is critical for increasing effective panicle per plant as well as the ratio of ear-bearing tillers in double-cropping super hybrid late rice. This contributes to a stable and high yield of rice.

Previously, it was believed that proper N supply at a late stage of development could improve the photosynthetic rate in the flag leaves of rice, delay aging of the flag leaves, and prolong photosynthetic time (Zhang et al., 2008; Sun et al., 2014). In this study, for double-cropping super hybrid late rice, N compensation at young panicle differentiation stage after N deficiency at tillering stage, the photosynthetic rate significantly (*P* < 0.05) increased in plants with normal dose N application (CK2) and double dose N compensation (T1), furthermore, Pn was maintained at a high level until a late stage of compensation. These results demonstrate outstanding compensation for N deficiency, which supports the production of photosynthates at late growth stages of rice. The results of this study are consistent with previous reports about the enhancement of Pn in the flag leaves of rice via N fertilizer (Zhang et al., 2008; Sun et al., 2014). NR and GS are key enzymes for the N metabolism in plants. Li et al. (2007) showed that activities of NR and GS in rice after resumption of N application were higher than those under N starvation. This study showed that double-cropping super hybrid late rice with N deficiency at tillering stage and N compensation at young panicle differentiation stage, activities of NR and GS were increased to some extent in plants with normal dose N application (CK2) and double N compensation (T1), compared with CK0, which plants N fertilizer was not supplied during all stages of growth (blank control). Consistent with a previous study (Li et al., 2007), this study indicates that compensation for N deficiency promotes N uptake and assimilation at later growth stages, thus improving N use efficiency in rice. In addition, it is believed that the regulation of growth, development and metabolism in plants are closely associated with endogenous hormones. Hormone regulation is mediated through the coordination of plant endogenous hormones, and a balance between hormones is sometimes more important than the action of a single hormone. The homeostasis of hormones controls the metabolism of nutrients, including nucleic acids, proteins, and soluble carbohydrates, thus influencing plant growth (Chen et al., 2013; Albacete et al., 2014; Hisano et al., 2016; Verbon and Liberman, 2016). In this study, ABA content was maximal in CK0 after N deficiency at the tillering stage and N compensation at young panicle differentiation stage. ABA contents in T1 and CK2 were significantly (*P* < 0.05) lower than in CK0 and CK1. Moreover, after N deficiency and compensation treatments, the ratio of total growth-promoting hormones (GA_3_ + IAA + ZR) to the growth-inhibitory hormone (ABA) was significantly (*P* < 0.05) higher in T1 and CK2 compared with CK0 and CK1. These results indicate the importance of the coordination of endogenous hormones, and also demonstrate that N compensation at young panicle differentiation stage is critical for the maintenance of a high ratio of pro- to anti-growth hormones in double-cropping super hybrid late rice for the improvement of yield.

Generation and accumulation of biomass in crops is the basis of yield, and the increase in the production of dry matter is the most direct approach to increase yield (Hasegawa, 2003). Farmers typically focus more on the base-tiller N fertilizer and overlook the importance of panicle-grain fertilizer. This practice promotes biomass accumulation during the early growth phase, but impedes biomass accumulation at later growth phases. It has been demonstrated that the yield of rice depends on the photosynthetic capacity from the heading stage to the milk-maturation stage, which means that more produced dry matter leads to a higher yield (Sui et al., 2013). In this study, double N compensation at young panicle differentiation stage after N deficiency at tillering stage (T1) caused maximal dry weigh and biomass per plant at maturation stage, ensuring maximal yield in this treatment group. This result demonstrates a compensatory effect of double-amount N at young panicle differentiation stage for N deficiency at the tillering stage in the double-cropping super hybrid late rice. Chen et al. (2015a,b) found that adequate N supply from tillering stage to heading stage, and particularly at young panicle differentiation stage, as well as control the application of N fertilizer after milk-maturation, could improve N use efficiency to a certain extent in super hybrid rice. In this study, for an equal amount of N supply in double-cropping super hybrid late rice, N uptake and utilization efficiency in plants that received a double N compensation after N deficiency (T1) was slightly higher (*P* > 0.05) than in plants with constant N supply throughout all stages (CK1). AE_N_ refers to the amount of yield increased per unit N fertilizer, reflecting the economic performance of N fertilizer in terms of grain production. AE_N_ in plants with N deficiency at tillering stage and compensation of normal (CK2) or double N compensation (T1) at young panicle differentiation stage were higher than in CK1, but there were no significant difference. PE_N_ reflects the capacity of converting N fertilizer into economic production. PE_N_ in CK2 and T1 was increased; however, it did not significantly differ from that in CK1. With an equal amount of total N supply, PE_N_ was higher after double N compensation compared with constant N supply, and HI_N_ and PFP_N_ showed the same pattern. In the double-cropping super hybrid late rice, double N compensation at young panicle differentiation stage after N deficiency at tillering stage was actually the postponement of N application with a larger panicle-grain N supply. This corresponds with the current practice of postponed N application, reduced fraction of base fertilizer, and panicle-grain N-oriented N application (Chen et al., 2011; Jiang et al., 2011). Overall, N compensation at young panicle differentiation stage after N deficiency at the tillering stage increases N use efficiency and reduces N waste. During cultivation, using appropriate panicle-grain N fertilization could thus optimize N use efficiency in double-cropping super hybrid late rice.

## Conclusion

In double-cropping super hybrid late rice, N compensation at young panicle differentiation stage after N deficiency at tillering stage resulted in yield compensation in plants. The yield per plant of T1 presented an equivalent compensatory effect, and CI was 1.12 and 1.05 for 2014 and 2015, respectively. Double N compensation (T1) showed a comparable yield per plant with constant N supply throughout all growth stages (CK1). N compensation led to superiority in the number of effective panicle per plant, increased activities of N metabolism-related NR and GS, decreased the content of endogenous growth-inhibitory hormone (ABA) in the leaves, increased the ratio of endogenous growth-promoting hormones (GA_3_ + IAA + ZR) to growth-inhibitory hormone (ABA), enhanced photosynthesis, and increased the number of tillers per plant. Adequate N supply at young panicle differentiation stage may improve AE_N_, RE_N_, HI_N_, PE_N_, and PFP_N_ in double-cropping super hybrid late rice. Consequently, the young panicle differentiation stage is an effective compensatory stage for prior N deficiency in double-cropping super hybrid late rice.

## Author Contributions

QX and GT designed and participated in all experimental procedures, performed data analysis, and drafted the manuscript. LZ participated in the figure preparation, samples preparation, and partial writing. HH participated in the figure preparation. XC supervised the study and critically revised the manuscript. All authors read and approved the final manuscript.

## Conflict of Interest Statement

The authors declare that the research was conducted in the absence of any commercial or financial relationships that could be construed as a potential conflict of interest.

## Abbreviations

ABA: abscisic acid
AE_N_: N agronomic efficiency
CI: compensation index
ELISA: enzyme-linked immunosorbent assay
GA_3_: gibberellin A_3_
GS: glutamine synthetase
HI: harvest index
HI_N_: N harvest index
IAA: auxin
N: nitrogen
NR: nitrate reductase
PE_N_: N physiological efficiency
PFP_N_: N partial factor productivity
Pn: net photosynthetic rate
RE_N_: N recovery efficiency
ZR: trans-zeatin-riboside
